# Dysbindin-1, BDNF, and GABAergic Transmission in Schizophrenia

**DOI:** 10.3389/fpsyt.2022.876749

**Published:** 2022-06-22

**Authors:** Rachel Jun, Wen Zhang, Nicholas J. Beacher, Yan Zhang, Yun Li, Da-Ting Lin

**Affiliations:** ^1^Intramural Research Program, National Institute on Drug Abuse, National Institutes of Health, Baltimore, MD, United States; ^2^National Institute on Drug Dependence and Beijing Key Laboratory of Drug Dependence, Peking University, Beijing, China; ^3^Department of Zoology and Physiology, University of Wyoming, Laramie, WY, United States

**Keywords:** schizophrenia, dysbindin-1, BDNF, GABAergic transmission, vesicular trafficking, activity-dependent release

## Abstract

Schizophrenia is a psychiatric disorder characterized by hallucinations, anhedonia, disordered thinking, and cognitive impairments. Both genetic and environmental factors contribute to schizophrenia. Dysbindin-1 (*DTNBP1*) and brain-derived neurotrophic factor (*BDNF*) are both genetic factors associated with schizophrenia. Mice lacking *Dtnbp1* showed behavioral deficits similar to human patients suffering from schizophrenia. DTNBP1 plays important functions in synapse formation and maintenance, receptor trafficking, and neurotransmitter release. DTNBP1 is co-assembled with 7 other proteins into a large protein complex, known as the biogenesis of lysosome-related organelles complex-1 (BLOC-1). Large dense-core vesicles (LDCVs) are involved in the secretion of hormones and neuropeptides, including BDNF. BDNF plays important roles in neuronal development, survival, and synaptic plasticity. BDNF is also critical in maintaining GABAergic inhibitory transmission in the brain. Two studies independently showed that DTNBP1 mediated activity-dependent BDNF secretion to maintain inhibitory transmission. Imbalance of excitatory and inhibitory neural activities is thought to contribute to schizophrenia. In this mini-review, we will discuss a potential pathogenetic mechanism for schizophrenia involving DTNBP1, BDNF, and inhibitory transmission. We will also discuss how these processes are interrelated and associated with a higher risk of schizophrenia development.

## Introduction

Schizophrenia is a neurodevelopmental disorder with a life-time prevalence of ~0.4% (1). It is characterized by positive symptoms (presence of auditory and visual hallucinations, delusions, disorganized behaviors), negative symptoms (loss of motivation, anhedonia, and blunted affect), and cognitive deficits (impairments in learning and problem solving) (2, 3). A combination of different genetic and environmental factors can increase the risk for schizophrenia (4). Studies have shown that schizophrenia has a heritability of ~80%, and twin studies established that genetics play an important role in the development of schizophrenia (5, 6). Further studies suggest significant associations between individuals with high schizophrenia polygenic risk scores and comorbidity with cognitive disorders, respiratory illness, and digestive diseases (7). Genetic influences to the development of schizophrenia are numerous and complex. In this mini-review, we will summarize evidence for the dysfunctions in dysbindin-1 (DTNBP1), a protein coding gene regularly implicated in schizophrenia as well as brain-derived neurotrophic factor (BDNF), and GABAergic circuit function in relation to schizophrenia.

## DYSBINDIN-1

Dysbindin-1 (DTNBP1) is a protein encoded by dystrobrevin-binding protein 1 gene (*DTNBP1*), located at chromosome 6, position 22.3 (8). It is a coiled-coil-containing protein and found in various brain regions (8, 9). A study on Drosophila's neuromuscular junction using electrophysiological screening revealed that DTNBP1 was required for presynaptic retrograde and homeostatic regulation of neurotransmission downstream or independently of calcium influx (10). Another electrophysiological study reported that DTNBP1 loss led to decreases in readily releasable pool in the calyx of Held synapses and could be related to the cognitive impairment in schizophrenia (11). In addition, a recent study has shown that DTNBP1 plays an important part in the axonal mitochondrial movement which further affects calcium homeostasis in presynaptic terminals (12). The null protein mutation of *Dtnbp1* in *sandy* (*sdy*) mice (13) displayed schizophrenia-like behaviors and deficits in dopaminergic, glutamatergic, and GABAergic neurotransmission (14–21). Patients with schizophrenia have significantly lower levels of *DTNBP1* mRNA in the dorsolateral prefrontal cortex, hippocampus, and nucleus accumbens compared to healthy controls (22). In the post-mortem brain, presynaptic DTNBP1 is reduced in synaptic terminals of hippocampal formations, which may contribute to cognitive deficits commonly seen in schizophrenia (9). Genetic studies have also provided evidence of *DTNBP1* impacting susceptibility to schizophrenia. Genome-wide association studies have identified multiple single nucleotide polymorphisms (SNPs) of *DTNBP1* as potential risk factors for schizophrenia (23). Several studies conducted in Japanese, Irish, and Chinese populations suggest that genetic variation in *DTNBP1* is associated with schizophrenia (8, 24, 25). DTNBP1 is also shown to be involved in initiating an immune response to environmental stimuli, which might explain the increased vulnerability of schizophrenia due to environmental impact in combination with genetic influence (26).

Studies have established the important role of DTNBP1 in intracellular protein trafficking, which affects various neuronal functions, including synapse formation and maintenance, receptor trafficking, and transmitter release (27–32). In neurons, DTNBP1 is located in the cytoplasm and can be assembled with several other proteins into a large protein complex, known as the biogenesis of lysosome-related organelles complex-1 (BLOC-1). BLOC-1 contains eight proteins: DTNBP1, cappuccino, pallidin, muted, snapin, and BLOS1-3 (33). In the central nervous system, BLOC-1 subunits, including DTNBP-1A, -1B, and -1C, are found in multiple brain regions, including hippocampal formation (HF) and are associated with synaptic vesicles or postsynaptic densities (34, 35). Previous studies reported DTNBP-1A as playing an important role in neuron development and spine growth (36–40). The DTNBP-1B subunit is present in humans, but not in mice (34). In studies involving genetically engineered mice, DTNBP-1B forms aggresomes at perinuclear regions in order to separate aggregated proteins produced by misfolded protein (41, 42). However, in humans, DTNBP-1B is diffused within the neuronal nuclei and axon terminals (34). Both DTNBP-1B and -1C isoforms are reduced in schizophrenic HF (34, 35) and significant reduction of DTNBP-1C was found in dorsolateral prefrontal cortex in schizophrenia (43). An experiment involving *sdy* mice with mutations in both DTNBP-1A and -1C indicated that decreases in DTNBP-1C led to decreased hilar mossy cells of dendate gyrus (35). This indicates the role of DTNBP-1C in maturation of newborn neurons in the dendate gyrus in a BLOC-1 independent manner (35). In addition, systematic investigation of BLOC-1 genes in schizophrenia patients revealed a significant association between the *BLOC1S3* gene and schizophrenia (44). During embryonic and early postnatal development, BLOC-1 is expressed more abundantly (45), which implicates an important role of BLOC-1 during early-life neural development. Another role of the BLOC-1 complex is the biogenesis of melanosomes and platelet-dense granules through self-assembly and interaction with actin cytoskeleton (46). Actin polymerization complex is a necessary organelle for synaptic function, and the expression of this actin cytoskeleton was reduced in *DTNBP1*-deficient cells (47). Other studies have shown that DTNBP1 recruited BLOC-1 is important for the regulation of oxytocin, metabotropic glutamate receptor, synaptic NMDA receptors, serotonin transmission, activity-dependent synaptic vesicle recycling, and synaptic plasticity (17, 48–55). Disruption of the BLOC-1 complex can lead to changes in the formation of large dense-core vesicles (LDCVs), which are involved in the secretion of hormones and neuropeptides. Studies have shown that LDCVs mediate the release of monoamines (Serotonin, Dopamine, and Noradrenaline) and peptides, including substance P, BDNF, and oxytocin (56). Of BLOC-1 complex proteins, loss of DTNBP1 or muted both led to the enlargement of LDCVs, and loss of DTNBP1 alone led to reduced LDCV numbers in cells from mice while muted deletion did not change the LDCV number (57). The release of LDCVs in neurons is also activity-dependently regulated in the central nervous system. For example, deletion of Munc13, a classic presynaptic protein involved in anchoring and activity-dependent release of synaptic vesicles, led to a reduction of LDCV release (58). BLOC-1 also contributes to the activity-dependent vesicle release. For example, it is shown that mutations of pallidin, whose encoded protein directly binds with DTNBP1 in BLOC-1, disrupted activity-dependent synaptic vesicle recycling (54). Therefore, DTNBP1 plays an essential role in activity-dependent neurotransmitter release, and DTNBP1 downregulations or genetic variations may contribute to schizophrenia by decreasing the size of readily releasable pool of synaptic vesicles which regulates synaptic transmission.

## BDNF

Another gene known to be associated with schizophrenia is brain-derived neurotrophic factor (*BDNF*) located in chromosome 11p13 (59). BDNF has been known to play important roles in neuronal development, survival, and synaptic plasticity (60). A single nucleotide polymorphism rs6265 in *BDNF* gene, known as Val66Met, impacts intracellular trafficking and activity-dependent secretion of BDNF (61), which can subsequently lead to memory impairment (62). A case-control association study of the Han Chinese population revealed a positive correlation between rs6265 and schizophrenia (63).

DTNBP1 is involved in the secretion of BDNF from pyramidal neurons in the cortex (64). Super-resolution imaging showed that DTNBP1 was located close to BDNF in the cytoplasm of neurons, possibly on LDCVs (65). Deletion of *Dtnbp1* in primary cultured neurons did not change spontaneous BDNF release but did change activity-dependent BDNF secretion (65, 66). These results suggest that DTNBP1 binds to LDCVs containing BDNF and regulates its activity-dependent secretion. The activity-dependent secretion of BDNF exerts a profound effect on synapse formation and maintenance as well as circuit function (67, 68). Activity-dependent production and secretion of BDNF exhibits a synapse-type specific effect on inhibitory synapses of hippocampal neurons. For example, disruption of activity-dependent transcription of BDNF selectively affects soma-targeting GABAergic synapses of hippocampal CA1 neurons, but not other types of GABAergic synapses or excitatory synapses of these neurons (69). This study suggests that activity-dependent BDNF production and its subsequent secretion from pyramidal neurons selectively affects the maintenance of those soma-targeting inhibitory synapses. Consistent with synapse-specific regulation by the activity-dependent secretion of BDNF, deletion of *Dtnbp1* in mouse prefrontal cortex (PFC) pyramidal neurons exclusively affected GABAergic inhibitory synaptic transmission, but not excitatory transmission (65), which has also been observed in cultured neurons (66). The resulting reduction in inhibition showed similar synapse-type specificity, as those located on the soma of pyramidal neurons were most affected. In contrast, GABAergic synapses targeting distal dendrites showed normal functions (65). The selective deletion of DTNBP1 also induced a behavioral deficit in pre-pulse-inhibition (PPI) that was rescued by direct administration of BDNF into the affected brain region (65). PPI deficiency is useful for modeling schizophrenia in animals (70) and has been used for decades to assess pharmaceutical efficacy in human patients suffering from schizophrenia (71). PPI behavioral deficits that were reversed by local infusion of BDNF in *Dtnbp1* knockout mice with schizophrenia-like behavior underscores the importance of DTNBP1 in regulating BDNF secretion and its role in the development and treatment of schizophrenia (65).

## GABAergic Transmission

**Figure 1:**
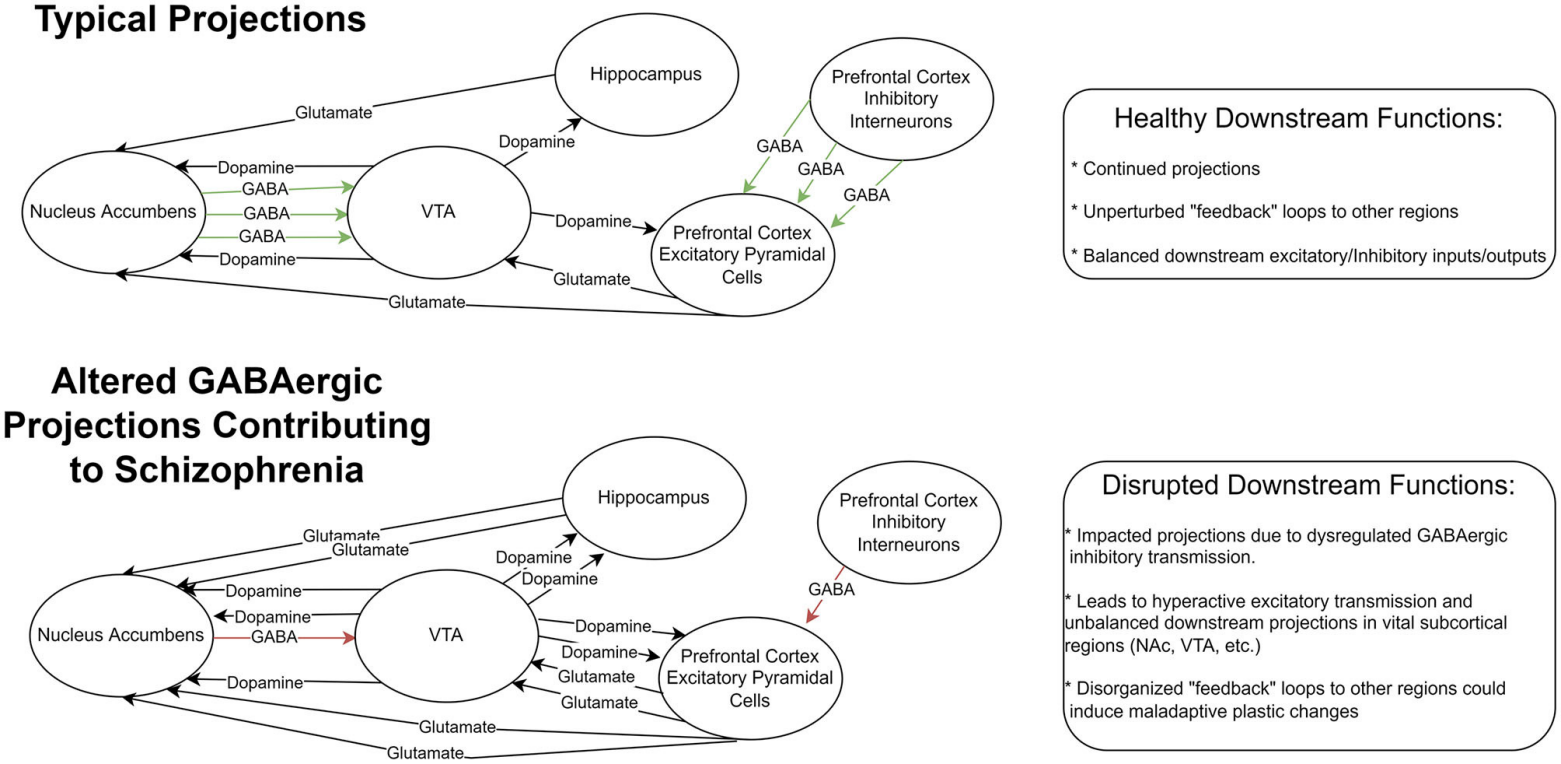
Representation of GABAergic circuitry in prefrontal cortex. Top row: A sample diagram depicting typical projections in a healthy system with functioning feedback loops and balanced excitatory and inhibitory inputs and outputs. Botom row: a sample diagram of this system altered by GABAegic cell changes seen in Schizophrenia. This altered system includes reduced inhibitory GABAergic signaling resulting in downstream hyperactive excitatory transmission. These could result in disrupted feedback systems and maladaptive plastic changes in vital subcortical regions.

The balance between excitatory and inhibitory transmission in the brain is vital for normal cognitive function. Studies have shown that dysfunction in GABAergic inhibitory circuits can lead to impaired cognition (72) and may contribute to schizophrenia (Figure 1). GABAergic interneurons are a heterogeneous group of neurons often categorized into at least three groups: parvalbumin-positive (PV+), somatostatin-positive (SST+), and 5-hydroxytryptamine 3a receptor-positive (5HT3R+) interneurons (73, 74). PV+ interneurons are one of the most abundant GABAergic interneurons in the cortex, comprising roughly 30% of interneurons in the cortex. PV+ neurons express parvalbumin, a Ca^2+^-binding protein in the cytoplasm, which could regulate the short-term plasticity of PV+ neurons (75). The majority of PV+ neurons emerge from Medial Ganglionic Eminence during development and migrate to the cortex (76). Mature PV+ neurons showed extensive axon arbors and connections to adjacent pyramidal neurons, which are considered non-selective (77). The axons of PV+ neurons mostly target soma and axon initial segment regions of the post-synaptic neurons; The majority of PV+ neurons show a high-frequency firing pattern when excited (76). These properties combined with their unique axon terminal location render PV+ neurons the major inhibitory driving force in the cortex (76). Moreover, PV+ neurons are also interconnected *via* electrical synapses, or gap junctions, making them more capable of synchronizing the inhibition of adjacent neurons (78). Of the other types of GABAergic interneurons, SST+ neurons mainly target the dendrites and soma of post-synaptic neurons, while 5HT3R+ neurons more likely target the distal dendrites of post-synaptic neurons (79, 80). The axon targeting specificity of these interneurons, together with differences in synaptic strength, enables diverse functions of neuronal micro-circuits. For example, in a simple circuit configuration of PV+, SST+, and excitatory neurons, the overall effect of SST+ neuron activities on excitatory neurons could be either inhibition or dis-inhibition when the microcircuit was located in different cortical regions (81, 82).

GABAergic transmission is regulated by the activity of glutamic acid decarboxylase (GAD) that participates in GABA synthesis (72). Several studies indicated that decreased expression of 67 kD isoform of GAD (GAD67) is associated with schizophrenia and bipolar disorders (83, 84). Analysis of GAD immunoreactivity in post-mortem brains from schizophrenic patients also reveal reduced GAD in patients compared to controls, indicating GABAergic dysfunction in schizophrenia (85). Interestingly, DTNBP1 emerges as one of the candidates for regulating inhibitory synapse strength. *In vitro* recordings of *Dtnbp1* deficient mice revealed significant decreases in GABAergic transmission at both pre- and post-synaptic levels and decreased parvalbumin markers (86). Deletion of DTNBP1 specifically in pyramidal neurons reduced the soma-targeting inhibitory synapse density without alterations in dendritic inhibitory synapses or excitatory transmission (65). Consequently, application of BDNF into extracellular space rescued this type-specific reduction of inhibitory synapses (65). Taken together, these results indicate that DTNBP1 plays several important roles in regulating inhibitory transmission and pathogenic mechanisms of schizophrenia, at least in part *via* BDNF secretion.

## Discussion

*Via* regulating activity-dependent BDNF secretion, DTNBP1 could regulate both neural development and inhibitory circuit function. It is also likely that malfunctioning of other DTNBP1 related processes could induce impairments that lead to the development of schizophrenia. While it is not yet known exactly how DTNBP1 regulates the functionality of different neural circuits in brain regions involved in schizophrenia, more studies are necessary to identify whether such a circuit change contributes to its role in the pathogenesis of schizophrenia. Modern *in vivo* imaging techniques can be harnessed to study how these different biological markers contribute to schizophrenia in animal models. For example, mouse models displaying schizophrenia-like behavior can be imaged with miniaturized microscopes (miniscopes) (87–93) to examine longitudinal changes in activity of excitatory and inhibitory neurons in brain regions implicated in schizophrenia. Simultaneous optogenetic manipulations of circuitry are also possible using miniscopes (94, 95) to explore how *in vivo* manipulation of circuitry impacts behavior using animal models for schizophrenia. These tools will further advance schizophrenia research in animals and help develop potential therapeutic interventions in human patients.

## Author Contributions

RJ and WZ wrote the manuscript with inputs from NB, YZ, YL, and D-TL. All authors contributed to the article and approved the submitted version.

## Funding

This research was supported by the Intramural Research Program, National Institute on Drug Abuse, National Institutes of Health, and National Institutes of Health Grants 5P20GM121310, R61NS115161, and UG3NS115608 to YL, and National Key R&D Program of China (Nos. 2019YFA0706201 and 2021ZD0202900), National Natural Science Foundation of China (No. 32170960) to WZ.

## Conflict of Interest

The authors declare that the research was conducted in the absence of any commercial or financial relationships that could be construed as a potential conflict of interest.

## Publisher's Note

All claims expressed in this article are solely those of the authors and do not necessarily represent those of their affiliated organizations, or those of the publisher, the editors and the reviewers. Any product that may be evaluated in this article, or claim that may be made by its manufacturer, is not guaranteed or endorsed by the publisher.
